# Identification of New Vulnerabilities in Conjunctival Melanoma Using Image-Based High Content Drug Screening

**DOI:** 10.3390/cancers14061575

**Published:** 2022-03-19

**Authors:** Katya Nardou, Michael Nicolas, Fabien Kuttler, Katarina Cisarova, Elifnaz Celik, Mathieu Quinodoz, Nicolo Riggi, Olivier Michielin, Carlo Rivolta, Gerardo Turcatti, Alexandre Pierre Moulin

**Affiliations:** 1 Jules-Gonin Eye Hospital, University of Lausanne, 1004 Lausanne, Switzerland; katya.nardou@fa2.ch (K.N.); michael.nicolas@fa2.ch (M.N.); 2 Biomolecular Screening Facility, Ecole Polytechnique Fédérale de Lausanne (EPFL), 1015 Lausanne, Switzerland; fabien.kuttler@epfl.ch (F.K.); gerardo.turcatti@epfl.ch (G.T.); 3 Medical Genetics Unit, Centre Hospitalier Universitaire Vaudois (CHUV), 1011 Lausanne, Switzerland; katarina.cisarova@chuv.ch; 4 Institute of Molecular and Clinical Ophthalmology Basel, 4031 Basel, Switzerland; elifnaz.celik@iob.ch (E.C.); mathieu.quinodoz@iob.ch (M.Q.); carlo.rivolta@iob.ch (C.R.); 5 Department of Ophthalmology, University of Basel, 4056 Basel, Switzerland; 6 Department of Genetics and Genome Biology, University of Leicester, Leicester LE1 7RH, UK; 7 Experimental Pathology, Institute of Pathology, Lausanne University, 1011 Lausanne, Switzerland; nriggi74@gmail.com; 8 Oncology Department, Centre Hospitalier Universitaire Vaudois (CHUV), 1011 Lausanne, Switzerland; olivier.michielin@chuv.ch

**Keywords:** conjunctival melanoma, drug screening, kinase inhibitors, MAPK pathway, PI3K/mTOR pathway, polo-like kinase, cyclin dependent kinase, aurora-kinase, Hsp90, Tirbanibulin

## Abstract

**Simple Summary:**

We determined the sensitivity of several conjunctival melanoma cell lines to kinase inhibitors as well as a few other inhibitors using an image-based high throughput drug screening assay with 542 compounds. All cell lines demonstrated sensitivity to cell cycle inhibition, especially with polo-like inhibitors. The response to MAPK pathway inhibition was better in the presence of *BRAF^V600E^* mutations, while the response to PI3k/mTOR inhibition was better in the presence of the *NRAS^Q61L^* mutation. Our study uncovers a large panel of new vulnerabilities in conjunctival melanoma and establishes the background for the expansion of therapeutic options in the management of this tumor.

**Abstract:**

Recent evidence suggests that numerous similarities exist between the genomic landscapes of both conjunctival and cutaneous melanoma. Since alterations of several components of the MAP kinases, PI3K/mTOR, and cell cycle pathways have been reported in conjunctival melanoma, we decided to assess the sensitivity of conjunctival melanoma to targeted inhibition mostly of kinase inhibitors. A high content drug screening assay based on automated fluorescence microscopy was performed in three conjunctival melanoma cell lines with different genomic backgrounds with 489 kinase inhibitors and 53 other inhibitors. IC50 and apoptosis induction were respectively assessed for 53 and 48 compounds. The genomic background influenced the response to MAK and PI3K/mTOR inhibition, more specifically cell lines with *BRAF ^V600E^* mutations were more sensitive to BRAF/MEK inhibition, while CRMM2 bearing the *NRAS^Q61L^* mutation was more sensitive to PI3k/mTOR inhibition. All cell lines demonstrated sensitivity to cell cycle inhibition, being more pronounced in CRMM2, especially with polo-like inhibitors. Our data also revealed new vulnerabilities to Hsp90 and Src inhibition. This study demonstrates that the genomic background partially influences the response to targeted therapy and uncovers a large panel of potential vulnerabilities in conjunctival melanoma that may expand available options for the management of this tumor.

## 1. Introduction

Conjunctival melanoma (CJM) most frequently arises from proliferation of atypical melanocytes localized in the conjunctiva (Conjunctival Intraepithelial Neoplasia, C-MIN/Primary Acquired Melanosis, PAM). A smaller proportion of conjunctival melanoma develops from preexisting conjunctival nevi or de novo. CJM is associated with a high recurrence rate and a significant mortality, affecting 25–35% of the patients at 10 years [1,2]. Similarly to cutaneous melanoma, the incidence of conjunctival melanoma, although rare, is increasing [3,4,5,6].

CJM not only shares clinical characteristics with cutaneous melanoma such as lymphatic metastatic spread, but the genomic background also shares numerous similarities with cutaneous melanoma. The genomic landscape is characterized by a high tumor somatic mutational burden and a predominance of C > T transitions, suggesting a role for UV light in the development of this tumor [7,8,9]. Our investigations as well as others revealed that driver mutations in *NF1* predominate (50–33%), followed by mutations in *BRAF* (46–29%) and *NRAS* (26–11%) [8,10,11,12,13,14]. These investigations suggest that the four molecular categories identified in cutaneous melanoma (*BRAF^mut^*, *NRAS^mut^*, *NF1^mut^*, Triple WT) [15] exist in the conjunctiva, but their proportions differ. Recently, inactivating mutations in *ATRX*, often co-occurring with mutation in *NF1* in tumors arising in the tarsal conjunctiva have been reported in 17 out of 68 conjunctival melanomas [16]. Both *TERT* promoter mutations identified in up to 54% of the cases and *NRAS* mutations have been associated with metastatic disease [14,16]. Mutations in *CTNNB1*, coding for β-Catenin, were recently identified in 17% of the cases [13]. Rare mutations include events in *RET*, *TP53*, *C-KIT*, *ARID2*, *TET2*, *CDKN2A*, *MAPK2*, *RAC1*, *MET* and even in genes mutated in uveal melanoma such as *SF3B1*, *GNAQ,* and *GNA11* [8,13,14,16]. The most common aneuploidy observed in conjunctival melanoma is chromosome 6p gain, reported in 61% of the cases [17]. Other gains include chromosomes 1q, 3p, 7p, 7q, 8q, 10q, 11p, 11q (encompassing the *CCND1* gene coding for cyclin D1), 12p, 13q, 14p, and 17q gains, while losses have been described in chromosomes 1p, 3q, 4q, 6q, 8p, 9p (encompassing the *CDKN2A* gene), 12p,14p, 17q, and 19 [8,11,17]. Interestingly, 10q losses have been associated with metastatic disease [17]. The combination of specific mutations and aneuploidies can lead to alterations of several signaling pathways, mostly affecting MAP kinase, Hippo, Wnt, Notch, p53, cell cycle, and pi3K [8]. In tumoral tissues of conjunctival melanomas, we and others have previously reported the activation of Mitogen-Activated Protein Kinase (MAPK) and possibly phosphoinositide 3-kinase (PI3k)/mTOR pathways [18,19]. In vitro, a sensitivity of CJM cell lines to BRAF (Vemurafenib), MEK (Trametinib, Binimetinib), and PI3/mTOR (MEK-2206, Dactolisib, Pictilisib) inhibition has been demonstrated, the sensitivity partially depending upon the presence of *BRAF^V600E^* or *NRAS^Q61L^* mutations [18,19].

Probably due to the rarity of CJM, reports documenting the use of BRAF inhibitors or MEK inhibitors in the management of this tumor are limited to less than 10 cases, with variable success [20]. In CJM and in cutaneous melanoma, BRAF and MEK inhibitors have mainly been used in a metastatic setting. In CJM, the successful use of a BRAF inhibitor or a combination of BRAF and MEK inhibitors to treat non-metastatic recurrent CJM have been described in two patients [20,21]. In cutaneous melanoma, the combination of two anti-PD1 checkpoint inhibitors for the treatment of advanced disease recently led to a significant improvement in 5-year survival rate, reaching 52% [22]. As the response to checkpoint inhibitors appears to be correlated with the tumor mutation burden [23], these inhibitors have also been used in a few patients with conjunctival melanoma with encouraging preliminary results [20]. However, in large studies of cutaneous melanoma, a proportion of patients either does not primarily respond to checkpoint inhibitors or ultimately develops late relapse and resistance [22,24,25,26]. Checkpoint inhibitors can also be associated with significant adverse effects and grade 3 or 4 toxicities [27,28]. Moreover, emerging data suggest that prolonged use of either targeted therapy [29] or checkpoint inhibitors [30] can be associated with the selection of resistant tumor cells or the establishment of a permissive tumor microenvironment. In that sense, the identification of alternative therapeutic options and targets for the management of advanced CJM is of primary importance.

Based upon our identification of alterations of signaling pathways in CJM, we aimed to assess the response of CJM cell lines with different genomic backgrounds to inhibition of these pathways. We, therefore, designed a high content drug screen of more than 500 compounds predominantly including tyrosine kinase inhibitors. Our study revealed previously unknown susceptibilities to multiple targets, and a genotype-dependent response to MAPK, PI3K/mTOR, as well as cell-cycle inhibitors, expanding the therapeutic options for the management of advanced CJM.

## 2. Material and Methods

### 2.1. Cell Lines

Conjunctival Recurrent Malignant Melanoma-1 cells (CRMM1; RRID: CVCL_M593), Conjunctival Recurrent Malignant Melanoma-2 cells (CRMM2; RRID: CVCL_M594), and CM2005.1 (RRID: CVCL_M592) were grown at 37 °C, with 5% CO_2_ in a medium composed of Ham’s F-12 (Kaighn’s) without phenol red (cat n° 21127-022, Gibco, Thermo Fischer Scientific, Waltham, MA, USA), supplemented with 10% Fetal Bovine Serum FBS (cat. n° F7524, Sigma-Aldrich, Burlington, MA, USA), antibiotic antimycotic 100× solution (cat. n° A5955 Sigma-Aldrich) as previously described [18]. CRMM1, CRMM2, and CM2005.1 were previously kindly provided by Martine Jager, University of Leiden. Cell passages were performed at confluency using Trypsin-EDTA (0.05%) (cat. n° 25300054, Thermo Fisher; Waltham, MA, USA).

### 2.2. Next Generation Sequencing and Variant Calling

Whole exome sequencing (WES) was performed in all three cell lines by using the Twist Comprehensive Exome Panel (Twist Bioscience, South San Francisco, CA, USA) on a HiSeq 4000 sequencer (Illumina, San Diego, CA, USA) raw reads were aligned to the UCSC hg19 genome reference [31] with BWA-mem [32]. All potential duplicate reads in BAM files were removed with the Picard MarkDuplicate tool (https://broadinstitute.github.io/picard/, accessed on 27 February 2022; Broad Institute, v.2.23.8). Base quality score recalibration was performed on the processed BAM files with GATK (v4.1) [33], BaseRecalibrator, and ApplyBQSR tools, followed by HaplotypeCaller to call SNVs and small INDELs in target regions.

### 2.3. Variant Annotation and Filtering

Variants were annotated with RefSeq [34], gnomAD [35], and ClinVar [36] databases by using ANNOVAR [37]. In-house scripts were used for annotating in silico prediction scores of the variants from MutScore [38] and MaxEntScan [39]. Variants were selected based on the following criteria: (i) AF < 0.1% in gnomAD exomes (all ethnicities together), (ii) significant genes associated with melanoma in My Cancer Genome, (iii) exonic or canonical splicing variants, (iv) intronic variants with predicted splicing effect using MaxEntScan, (v) variants submitted as pathogenic or likely pathogenic in ClinVar database. HGVS notations were checked using VariantValidator (https://variantvalidator.org/, accessed on 15 February 2022). Presence of variants in different cancer types was checked in COSMIC v95 (https://cancer.sanger.ac.uk/cosmic, accessed on 18 February 2022).

### 2.4. Drug Screening

The compounds screened were selected from different Ecole Polytechnique Fédérale de Lausanne (EPFL)’s Biomolecular Screening Facility (BSF) compounds libraries, generating a sub-collection of 489 kinase inhibitors and 53 other selected inhibitors (Supplementary Figure S1). Chemical integrity and purity were confirmed for each compound by Liquid Chromatography Mass Spectrometry prior to screening. Compounds (10 mM stock in DMSO) were pre-spotted in 384 well plates (cat. n° 353962, Falcon, Corning, Corning, NY, USA) using Echo acoustic dispensing (Labcyte Inc., San Jose, CA, USA), and cells were then seeded on top of the compounds (reverse mode) using a cell dispenser (Biotek, Agilent, Santa Clara, CA, USA), reaching a final concentration of 1 µM for each compound in 25 µL. The plates were incubated for 72 h. The cells were than labeled with CalceinAM and ethidium homodimer-1 according to the manufacturer’s protocol (Live/DEAD Viability/Cytotoxicity Kit for mammalian cells, cat. n° L3224, Thermo Fisher Scientific), followed by additional staining with Hoechst 33342 (Sigma-Aldrich, St. Louis, MO, USA) and were live imaged using an INCell Analyzer 2200 (GE Healthcare, Boston, MA, USA). Automated acquisition was performed in an environmental chamber (at 37 °C with 5% CO_2_) using a 4×/0.2 NA objective, allowing imaging of the full well surface in a single image, while still allowing precise segmentation of individual cells, nuclei, or regions of interest.

Dose–response curves were established for 53 compounds with concentrations ranging from 3000 to 0.1 nM using Echo acoustic dispensing, in the same conditions as for the primary screen.

We acknowledge the NCCR Chemical Biology supported by the SNSF for purchasing the BSF-EPFL Collections.

### 2.5. Apoptosis Assay

Apoptosis induction was assessed using Annexin V assay (cat. n° V13241, ThermoFischer, Waltham, MA, USA) with concentrations ≥ IC50 previously obtained grouped in three categories (100, 500 and 1000 nM) using Echo acoustic dispensing. Annexin V, Hoechst, and ethidium homodimer-1 stainings were performed in 96-well plates (PhenoPlate #6055302, Perkin-Elmer, Waltham, MA, USA) after 48 h of incubation and automated imaging was performed in the same conditions as for the primary screen, but using a 10×/0.45 objective.

For the entire screening assay, the dose–response curve and apoptosis assays were performed in duplicate. A preliminary validation for each assay and for each cell line was performed using the statistical Z’ approach [40], using plates composed of quarter-plates (384-well) or half-plates (96-well) with DMSO as negative control and quarter-plates (384-well) or half-plates (96-well) with Gambogic acid (primary screen and dose–responses) or Staurosporine (apoptosis assay) as positive control.

### 2.6. Automated Image Analysis Pipeline and Data-Analysis Workflow

All the images were analyzed using either pipelines in CellProfiler v4.2.1 (https://cellprofiler.org/citations/, accessed on 22 June 2021) [41] or custom-made macros in Fiji [42] and extracted data were processed either through the EPFL-BSF in-house LIMS or through workflows in Knime v4.5.1 (https://www.knime.com/, accessed on 20 January 2022) with the assessment of size, shape, and intensity features of all segmented objects. Each plate contained replicates of positive and negative controls (8 replicates or 32 replicates each for 96-well and 384-well plates, respectively), and all results were normalized according to the controls of the corresponding plate. Screen hit assignment was based upon the normalized values, a hit being defined as such if the value was 3 SD superior to the corresponding negative control for all replicates.

Depending on the assay, different features and calculated values were considered as readouts: for the primary screen and the dose responses, total cell counts (based on Hoechst staining) and dead-cell ratios (based on intensities of CalceinAM and Ethidium Homodimer-1 for each segmented cell) were utilized. For the apoptosis assay, total cell counts (based on Hoechst staining) and percentages of Annexin V-positive cells were considered (based on Annexin V intensity).

## 3. Results

### 3.1. Genomic Background

Mutations identified in all cell lines are summarized in Supplementary Table S1. WES revealed that CM2005.1 contained the *BRAF^V600E^* mutation as well as missense variants with uncertain significance in *NSD1* and *BRCA1*; however, no other clear-cut mutation was detected.

CRMM1 harbored the *BRAF^V600E^* mutation as previously published [18]. Interestingly, a probably damaging mutation occurring in the tyrosine kinase domain of the C-ROS oncogene (*ROS1*), ROS1*^P2161S^*, was also discovered, together with a frameshift mutation in *TET2* Methylcytosine Dioxygenase 2 (*TET2^p.Glu1459LeufsTer)^*). *ROS1^P2161S^* is predicted to be pathogenic by in silico tools. CRMM1 contained as well a *CDKN2A* c.193 + 5G > A mutation, affecting p14^ARF^, and a probably damaging mutation in the retinoblastoma gene, *RB1*^S648L^.

CRMM2 contained a *NRAS^Q61^*^L^ mutation as previously described [18], as well as a probably pathogenic mutation in the glycin rich domain of *ALK^G936E^*, previously reported in basal cell carcinoma [43] and skin melanoma. A mutation affecting the gene coding for cyclin D1 was also found in *CCND1^P287L^*. In vitro, P287L impairs the interaction between CCND1 and the AMBRA1 complex, which is responsible for ubiquitination and subsequent degradation of CCND1 [44].

### 3.2. Screening Assay Validation and Hits Assignment

**Figure 1:**
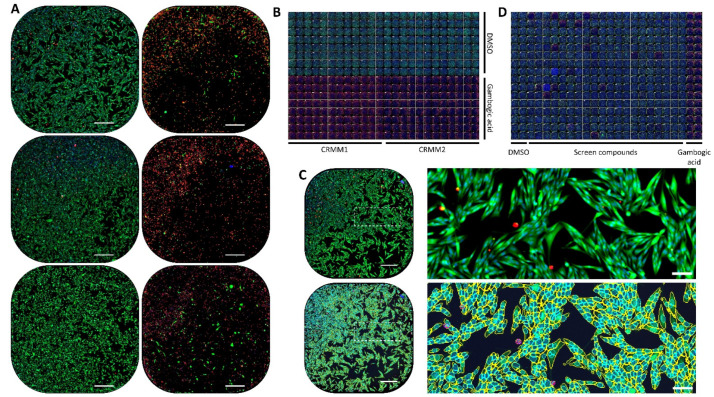
Live/Dead viability/cytotoxicity assay screen. (**A**) Example images of the control conditions for all 3 conjunctival melanoma cell lines. Upper row: CRMM1; middle row: CRMM2; lower row: CM2005.1. Left: DMSO; Right: Gambogic Acid. All nuclei are stained blue with Hoechst 33342, live cells, green with Calcein AM, and dead nuclei red with Ethidium homodimer-1. Objective 4×/0.2. The full well area is contained in a single image per well. Scale bar 500 μm. (**B**) Full plate snapshot of the Z’ plate validating the assay for CRMM1 and CRMM2 cell lines. All images of the plate, acquired in the same conditions as in (**A**), are montaged together. (**C**) Image segmentation performed with CellProfiler. DMSO control image of CRMM1 cells stained as in (**A**) (top row), and with the overlays of segmented regions (bottom row). Cyan: all nuclei. Yellow: live Calcein AM-positive cells. Pink: dead Ethidium homodimer-1-positive nuclei. Right: zoomed crop of the area highlighted by a dashed rectangle. Scale bars 500 µm (left) and 100 µm (right). (**D**) Full plate snapshot example of one of the screen plates with CRMM1 cells, composed of 32 replicates of each of the controls and 320 screen compounds, montaged together after imaging as in (**A**).

The Z’ scores obtained for each plate (mean: 0.92 for CM2005.1, 0.68 for CRMM1, 0.92 for CRMM2) were very good, validating each of the screening plates, and providing excellent screening windows (Figure 1).

In total, 60 hits (40 with score ≥ 0.1) were identified for CM2005.1, 49 hits (34 with score ≥ 0.1) for CRMM1, and 58 hits (48 with score ≥ 0.1) for CRMM2 (Supplementary Table S2). For all the three cell lines, the most potent inhibitors were cyclin dependent kinase (CDK) inhibitors: flavopiridol (Alvocidib), SB1317 (TG02, Zotiraciclib), a multikinase inhibitor targeting CDK (1, 2, 3, 5, 7, 9), Janus Kinase 2 (JAK2), Fm-Like tyrosine kinase 3 (FLT3), and dinaciclib (inhibiting CDK1, CDK2, CDK5, and CDK9). PIK-75, a PI3K inhibitor targeting the p110α catalytic subunit, was also very efficient.

### 3.3. Dose–Response Curves

Dose–response curves were established for 53 selected compounds (with a hit score ≥ 0.05 for at least one of the cell lines). All Z’ scores obtained for each cell line (means 0.70 for CM2005.1, 0.48 for CRMM1, 0.65 for CRMM2) allowed a good validation of the plates, providing results that could be further analyzed and fitted for IC50 determination. The results are summarized in Table 1.

Globally, the response to MAP kinase inhibition was better and stronger in cell lines with the *BRAF^V600E^* mutation than in CRMM2, bearing the *NRAS^Q61L^* mutation. CM2005.1 was the most sensitive cell line to MAPK pathway inhibition with IC50 below 50 nM for six MEK inhibitors and Dafrafenib. CRMM1 was also responsive with IC50 below 50 nM for five MEK inhibitors and PLX4720, while CRMM2 was less responsive with one IC50 below 50 nM for AZD8330 and for PLX4720. Among the MEK inhibitors, AZD8330 was associated with a stronger inhibition in all cell lines. Interestingly, downstream inhibition of ERK with Ravoxertinib was relatively potent in CRMM1, but inefficient in CRMM2. C-MET inhibitors were not as efficient as BRAF or MEK inhibitors in all cell lines.

The inhibition of the PI3K/mTOR axis was more efficient in CRMM2 than in CM2005.1 and CRMM1. More specifically, IC50s were lower in CRMM2 for five mTOR inhibitors, three PI3K inhibitors, and a dual PI3K/mTOR inhibitor, Omipalisib. In CRMM2, this dual inhibition of PI3K and mTOR was the most efficient. A response to Sirolimus was only observed in CRMM1.

Cell cycle inhibition was assessed with cyclin dependent kinase (CDK) inhibitors and with antimitotic polo-like kinase inhibitors and aurora kinase inhibitors. Overall, the inhibition was stronger in CRMM2. Dinaciclib, an inhibitor of CDK2, 9, 7, and 5, was associated with the lowest IC50 in all the cell lines. Importantly, no inhibitors of cyclin dependent kinase 4/6 (Abemaciclib, Ribociclib) were identified as hits in the three cell lines. RGB-286638 was effective in all cell lines, but its effects might also be attributed to the possible multikinase inhibition of GSK-3β, JAK2, and MEK1. Polo-like inhibitors were more effective in CRMM2 and CRMM1: in CRMM2, 4 out of 5 polo-like inhibitors were associated with IC50 below 100 nM and 2 out of 3 in CRMM1. Two aurora kinase inhibitors were effective in CRMM2 and Alisertib was associated with an IC50 of 32.7 nM.

Ganetespib, a heat shock protein 90 inhibitor, used in clinical trials for the treatment of non-small cell lung carcinoma with *EGFR*, *KRAS,* or *ALK* mutations, was effective in all cell lines with a stronger inhibition in CRMM2.

### 3.4. Apoptosis Assay

**Figure 2:**
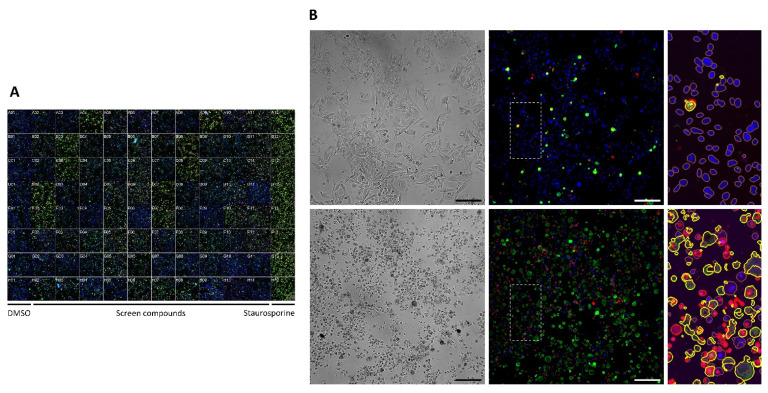
Annexin V apoptosis assay screen. (**A**) Full plate snapshot example of the Annexin V screen with CM2005.1 cells, composed of 8 replicates of each of the controls and 80 screen compounds, montaged together. All nuclei are stained blue with Hoechst, apoptotic cells green with Annexin V, and dead nuclei red with Ethidium homodimer-1. Objective 10×/0.45. The snapshot is composed of a single field of view out of the 9 fields acquired per well. (**B**) Example images of the control conditions for CM2005.1 cells. Top: DMSO. Bottom: Staurosporine. Left panel: brightfield images. Middle panel: fluorescent images acquired as in A. Right panel: zoomed crop of the area highlighted by a dashed rectangle, with an overlay of image segmentation performed by CellProfiler. Brown: all nuclei. Yellow: Annexin V-positive apoptotic cells. Red: dead Ethidium homodimer-1 positive nuclei. Scale bar 200 µm.

The Z’ scores obtained for each plate (CM2005.1:0.61, CRMM1: 0.73, CRMM2: 0.80) were again excellent and allowed a good discrimination of the Annexin V positive hits (Figure 2).

C-Met Inhibition by Tivantinib was not associated with induction of apoptosis in CM2005.1 and CRMM1. Apoptosis induction was observed in CM2005.1 with all MEK inhibitors tested and in CRMM2 with the six MEK inhibitors efficient in this line. In CRMM1, only Cobimetinib, AS703026, AZD8330, and TAK-733 triggered apoptosis, but Selumetinib, PD184352, and GDC-0094, despite low IC50, did not induce apoptosis.

Inhibition of the PI3K/mTOR pathway and cell cycle inhibition with CDKI was systematically associated with induction of conventional apoptosis when the cell lines were sensitive to the drugs, as demonstrated by the Annexin V assay.

Except for Rigosertib inducing apoptosis in all cell lines, apoptosis was not seen with polo-like kinase inhibitors in CM2005.1. Apoptosis induction was more frequently observed with polo-like kinase in CRMM2 and CRMM1.

Among the three checkpoints kinase inhibitors studied, only CHIR-24 was effective in all three cell lines and associated with apoptosis induction.

## 4. Discussion

Based upon our previous studies combining genomic and transcriptomic data as well as pathway activation in CJM, we assessed, in vitro, the response to kinase inhibition in the largest image-based high content drug screening study performed so far in this tumor. Our results revealed previously unknown susceptibilities to multiple drugs, establishing a framework for the selection of future targeted therapy in the management of advanced CJM.

Upstream inhibition of the MAPK kinase pathway with BRAF inhibitors PLX470 and Dabrafenib was, as expected, more efficient in cell lines with *BRAF^V600E^* mutations and the lower IC50 identified with Dabrafenib for CM2005.1 (44.16 nM) lays within the same range as previously reported for this drug [19]. PLX4720 was also very efficient in CRMM1 with an IC50 of 25.1 nM and apoptosis induction at the 100 nM concentration. However, the very low IC50 identified for CRMM2 with the *NRAS^Q61L^* mutation with PLX4720 is, however, surprising and might possibly be due to a non-specific toxic effect.

Our study confirms that MEK inhibitors are a treatment of choice for cell lines with *BRAF^V600E^* mutations. Among the 10 MEK inhibitors assessed, AZD8330, an ATP non-competitive MEK1/MEK2 inhibitor, appears to be the most efficient with the lowest IC50 and apoptosis induction in all the cell lines. However, in a phase 1 clinical study with AZD8330 in 82 patients including 18 cutaneous melanomas [45], only one partial response was observed and 26.8% of the patients had stable disease for more than 3 months, leading the authors to suppose that tumor penetration of AZD8330 might not have been sufficient in their study. Pimasertib, a non-competitive MEK1/MEK2 inhibitor, was also efficient in all our cell lines and a phase I dose-escalation study performed with this drug in 89 patients with metastatic melanoma revealed a complete response in a melanoma with the *NRAS* mutation, an objective response in 12.4% of the patients, and stable disease in 55% of the patients [46]. Although a good response was observed in our cell lines with TAK-733, an allosteric MEK1/MEK2 inhibitor, as previously observed in other cutaneous melanoma cell lines in vitro and in vivo [47], in a phase I clinical study in 41 patients including 12 with metastatic uveal melanoma and five with metastatic cutaneous melanoma, a modest response rate (5%) was observed with only two patients with cutaneous melanoma experiencing a partial response rate [48]. Several large phase 3 randomized studies in cutaneous melanoma with *BRAF^V600E^* mutations [49,50,51] revealed the advantages of a combination of MEK inhibitors (Cobimetinib or Trametenib) with BRAF inhibitors (Dabrafenib or Vemurafenib) over monotherapy with the BRAF inhibitor. This was demonstrated by an improved overall survival and reduction in secondary squamous carcinoma, resulting from combined therapy.

Downstream MAPK inhibition with ERK inhibitors was only effective in CRMM1 with low IC50 identified both for PD184352 and Ravoxertinib, without apoptosis induction. Antitumor activity has been demonstrated with Ravoxertinib in a phase I study in 47 patients with advanced solid tumors with stable disease in 34% and a partial response in two colon carcinomas with *BRAF^V600E^* mutations [52].

Inhibition of the PI3K/mTOR axis was more efficient in CRMM2 with *NRAS^Q61L^* mutation and Omipalisib, a dual inhibitor of the p110 subunit of Pi3K and the mTOR inhibitor, was associated with the lowest IC50 in our study. Our results are in line with previous data in *NRAS*-mutated skin melanoma cell lines where Omipalisib was the most potent inhibitor of the PI3K/mTOR pathway [53]. In a large phase I study including 170 patients with solid tumors, single use of Omipalisib had, however, a modest antitumor activity with an objective response of only 5% [54]. The combination of Omipalisib and MEK inhibitors significantly reduced tumor size in vivo in xenograft tumor models and was synergistic in vitro in *NRAS*-mutated skin melanoma cell lines [53]. This combined inhibition of Pi3K/mTOR and MEK inhibitors with Omipalisib and Trametinib was assessed in a phase 1b study in 69 patients with advanced solid tumors: the necessity to reduce doses due to frequent toxicities (skin rash 71%, diarrhea 61%) in 42% of the patients probably explained the limited antitumor activity observed with only one partial response and 12 patients with stable disease [55]. The efficiency of Sapanisertib, a third generation ATP competitive inhibitor of mTOR, has been demonstrated in vitro and in vivo in breast carcinoma [56], bladder carcinoma [57], and renal cell carcinoma [58]. Recently, in an expansion phase I study, manageable toxicity and preliminary antitumor activity were reported with this drug in renal cell carcinoma and endometrial carcinoma [59].

Inhibition of cellular division was more effective in CRMM1 and CRMM2 which grow faster than CM2005.1. Of note, CRMM1 harbors a *RB1*^S648L^ mutation and CRMM2 contains a *CCND1^P287L^* mutation that impairs Cyclin D1 degradation. Among cyclin dependent kinase inhibitor (CDK), Dinaciclib, a second-generation CDK inhibitor of CDK2, CDK9, CDK5, and CDK1, was the most potent with the lowest IC50. Our results are similar to those observed in vitro with several cell lines including skin melanoma cell lines where caspase 3 activation was noted in most of the cases [60]. In cutaneous melanoma cell lines, Dinaciclib-induced apoptosis was dependent on p53 signaling and expression both in vitro and in vivo [61]. The good response observed in our study with CM2005.1, CRMM1, and CRMM2 could possibly be linked the low level of p53 expression recently demonstrated in these cell lines [62]. In cutaneous melanoma [61], the effects of Dinaciclib correlated neither with the presence of *BRAS* or *NRAS* mutations, nor with CDK2 levels. In lung carcinoma cell lines, apoptosis induction by Dinaciclib was, however, stronger in the presence of a *KRAS* mutation [63]. Clinical results with Dinaciclib as a single agent have been variable: in phase II randomized clinical studies in breast carcinoma [64] and non-small cell lung carcinoma [65], clinical activity was limited to none to a few patients, while preliminary encouraging results with an overall response rate of 40% were found in a phase III study in patients with relapsed/refractory chronic lymphocytic leukemia [66]. Clinical trials using Dinaciclib as a single agent in the treatment of advanced cutaneous melanoma are ongoing. As a synergism was recently demonstrated in vitro with the combination of Dinaciclib and MEK inhibitor (Trametinib) in skin melanoma [67], it is possible that such combinations may be clinically relevant in the future for the treatment of advanced melanoma. Interestingly, the recent demonstration of increased immune infiltrate in xenograft tumor models treated with Dinaciclib and anti-PD1 checkpoint inhibitor along with the interferon type I gene signature induced in vitro by Dinaciclib [68], suggest that the combination of Dinaciclib and anti-PD1 monoclonal antibodies may potentiate the effect of anti-PD1 treatment and improve immune response.

Polo-like kinases are important regulators of cell division and Polo-Like kinase 1 has been involved in centrosome maturation, spindle assembly, mitosis entry, chromatin condensation, and cytokinesis [69]. Plk1 overexpression has been identified in many cancers [69] and Plk1 expression was increased in melanoma and metastatic melanoma compared to benign nevi [70,71]. Plk1 inhibition with Volasertib was successful at low concentrations similar to our study in melanoma cell lines in vitro with growth inhibition [71]. In this study, the authors further demonstrated an antitumor effect in vivo with apoptosis induction and p53 expression. The lower IC50 identified for CRMM2 with a *NRAS* mutation with Volasertib and other polo-like inhibitors can be correlated with the increased mitotic stress identified in *RAS* mutated cells and increased sensitivity to PLK inhibition [72]. In a phase I dose escalation study with volasertib as a single agent in 65 patients with advanced solid tumors including 12 melanomas, a partial response was seen in three patients including a patient with melanoma and stable disease was observed in 40% of the patients [73].

Aurora kinases play important roles during mitosis. More specifically, aurora kinase A (AKA) is involved in spindle assembly, centrosome maturation, and separation and cytokinesis [74]. AKA expression was found to correlate with tumor progression and poor prognosis [75], and its overexpression in general has been identified in several cancers and in cutaneous melanoma [75]. Inhibition of AKA in cancer cell lines resulted in disorganization of microtubule spindles and mitosis arrest followed by apoptosis [76]. In cutaneous melanoma cell lines, AKA inhibition resulted in growth arrest in vitro and in vivo and apoptosis induction in vitro [77]. Alisertib, an AKA inhibitor associated with low IC50 in CRMM2 in our study, induced growth arrest and apoptosis in several skin melanoma cell lines [75], including resistant melanoma cell lines [78]. In skin melanoma cell lines, MAPK activation was associated with upregulation of AKA mediated by promoter activation with FOXM1 [75]. The identification of MAPK kinase activation upon AKA kinase inhibition in skin melanoma cell lines also led to the suggestion of combining AKA inhibition with BRAF or MEK inhibitors to overcome potential resistance [78]. Evidence also suggests that the combination of AKA kinase inhibition might potentiate the response to immunotherapy: in one study, AKA inhibition in immunocompetent mice models with skin melanoma cell lines led to reduced tumor growth, upregulation of genes involved with immune response, as well as an increased T cell infiltrate [79]. In this study, combining T cell stimulation and AKA inhibition led to a significant tumor reduction compared to single treatment. In another study, Aurora kinases were found to be implicated in melanoma resistance to T-cell cytotoxicity and combined treatment with Aurora kinase B inhibitor and ipilimumab led to significant tumor reduction in vivo [80]. Clinically, Alisertib has been most effective as a single agent in patients with relapsed/refractory peripheral T cell lymphoma in a phase III study where a response rate of 33% was identified [81].

In our study Ganetespib, a heat shock protein 90 inhibitor was associated with IC50 below 100 nM in two cell lines and apoptosis induction in all cell lines. Heat shock protein 90 (Hsp90) has been implicated in the assembly of multiple chaperone complexes, regulating the stability and function of client proteins such as wild type c-RAF [82], mutant BRAF [83], mutant EGFR [84], as well as the EML4-ALK fusion protein in lung cancer [85]. Inhibition of Hsp90 results in depletion of client proteins and alterations of pathways important for survival [86]. In skin melanoma cell lines, Ganetespid induced downregulation of the MAPK signaling pathway both in *BRAF*- and *NRAS* mutated cell lines [87]. Ganetespid was further associated with in vitro and in vivo growth arrest, and apoptosis induction was found in vitro. The combination of Ganetespid and a MEK inhibitor, TAK-733 (also very effective in conjunctival melanoma cell lines in our study), allowed significant growth reduction in an in vivo model of *BRAF* resistant melanoma cell lines [88]. Evidence also suggests that Ganetespid might potentiate the effect of immunotherapy in cutaneous melanoma: Ganetespid increased interferon response genes in patient derived melanoma cell lines and the combination of Ganetespid and ipimilumab significantly reduced tumor size in vivo [89]. In this study, the combination of Ganetespid and Ipimilumab also increased the number of T CD8+ within the tumor, while decreasing T-Reg. Another Hsp 90 inhibitor, XL888, also showed preclinical activity in skin melanoma and promising results were observed in phase I with objective response in 75% (15/20) patients with skin advanced melanoma treated with Vemurafenib and XL888 [90]. Clinical trial in patients with advanced *BRAF*-mutated melanoma assessing the combination of XL888 and Vemurafenib and/or Cobimetinib are ongoing.

KX2-391, Tirbanibulin, was also very efficient in CRMM2. Tirbanibulin reversibly binds β-tubulin and inhibits tubulin polymerization [91]. In breast cancer cell lines, Tirbanibulin impaired normal mitosis, leading to mitotic catastrophe [92], growth arrest in vitro and in vivo, as well as decreased Src signaling. Apoptosis, as seen in our situation with all conjunctival melanoma cell lines, was demonstrated in breast cancer lines [93] and ovarian cancer cell lines [94]. The potency of Tirbanibulin has been reported in several cell lines including cutaneous melanoma cell lines. Tirbanibulin has been evaluated in the management of actinic keratosis with a complete clearance of actinic keratosis at 2 months in 49% of the cases [95].

The use of 2D cultures of established conjunctival melanoma cell lines is a limitation of our study. Despite several attempts, patient-derived short-term 3D cultures in spheroids from conjunctival melanoma have, so far, not been successful in our research. We nevertheless believe that the results presented in our study are valid as the genomic background of the cell lines used reflects the oncogenic drives observed in conjunctival melanoma.

## 5. Conclusions

In the largest drug screening performed so far in CJM, we assessed the response of CJM cell lines with different genomic background to kinase inhibitors targeting multiple dysregulated pathways. We believe that the identification of new vulnerabilities not only establishes new mechanistic research perspectives, but also uncovers new opportunities for the management of either locally advanced primary CJM or metastatic CJM. The combination of cycle cell inhibitors with either checkpoints inhibitors or MEK inhibitors appears notably as a promising option of overcoming tumor resistance and increasing tumor response. Our manuscript also highlights the necessity of fully characterizing the genomic background of all tumor cells, in order to optimize the therapeutic response.

## Figures and Tables

**Table 1:** IC50 and apoptosis.

Drug	Other Name	Drug Class	CM2005.1 IC50 nM	CM2005.1 Apoptosis	CRMM1 IC50 nM	CRMM1 Apoptosis	CRMM2 IC50 nM	CRMM2 Apoptosis
Tivantinib ^1^		MET I	452.25	N	756.47	N	390.85	N
Tivantinib ^2^		MET I	352.81	N	843.04	N	378.37	Y
PLX4720		BRAF I	ns		25.6	Y	5.72	N
Dabrafenib		BRAF I	44.16	Y	988.87	Y	ns	
RAF-265	CHIR-265	BRAF I	1012.16	nt	1001.5	nt	1048.7	nt
Trametinib ^3^		MEK I	475.61	Y	4.71	N	ns	
Trametinib ^4^		MEK I	22.09	Y	ns		107.1	Y
PD0325901	Mirdametinib	MEK I	24.52	Y	0.73	N	431.59	Y
Cobimetinib		MEK I	44.42	Y	349.57	Y	488.53	Y
AS703026	Pimasertib	MEK I	52.4	Y	292.37	Y	439.79	Y
AZD8330		MEK I	12.5	Y	103.94	Y	62.94	Y
TAK-733		MEK I	31.85	Y	640.35	Y	134.27	Y
AZD6244	Selumetinib	MEK I	229.98	Y	1.85	N	ns	
PD318088		MEK I	261.88	Y	247.98	N	ns	
PD184352		MEK I	748.43	Y	33.64	N	ns	
GDC-0994	Ravoxertinib	ERK I	816.06	Y	32.25	N	ns	
VRT752271	Ulixertinib	ERK I	ns		294	N	ns	
A-674563		AKT I	1001.89	Y	630.25	N	ns	
GSK2126458	Omipalisib	PI3k/mTOR	883.6	Y	657.42	Y	164.6	Y
PIK-75		PI3K I	389.79	Y	701.89	Y	439.64	Y
PI-103		PI3K I	ns		ns		ns	
PF-04691502		PI3K I	948.41	Y	ns		648.29	Y
GDC-0980	Apitolisib	PI3K I	938.46	Y	ns		871.75	Y
BKM120	Buparlisib	PI3K I	3000	nt	1220.8	nt	867.2	nt
Rapamycin	Sirolimus	mTOR I	ns		115.15	Y	ns	
INK-128	Sapanisertib	mTOR I	536.15	Y	427.96	Y	219.17	Y
AZD8055		mTOR I	603.26	Y	769.45	Y	322.95	Y
WYE-125132		mTOR I	604.89	Y	778.8	Y	286.97	Y
AZD2014	Vistusertib	mTOR I	ns		ns		740.23	Y
Torin 2		mTOR I	ns		558.31	Y	419.91	Y
Dinaciclib		CDK I	67.66	Y	113.84	Y	38.82	Y
SNS-032		CDK I	ns		ns		609.51	Y
Flavopiridol	Alvocidib	CDK I	594.29	Y	427.26	Y	540.87	Y
RGB-286638		CDK I	361.93	Y	366.39	Y	259.59	Y
Volasertib		PLK I	108.5	N	37.14	N	29.32	Y
GSK461364		PLK I	799.39	N	58.1	Y	12.77	Y
ON-01910	Rigosertib	PLK I	363.93	Y	528.75	Y	55.24	Y
NMS-1286937	Onvansertib	PLK I	105.48	N	297.29	Y	36.6	N
HMN-214		PLK I	340.78	N	952.54	Y	775.01	Y
Alisertib		Aurora K I	ns		467.01	N	32.77	Y
MLN8054		Aurora K I	213.36	N	975.24	N	335.6	N
Ganetespib		Hsp 90 I	94.41	Y	282.74	Y	38.65	Y
AT9283		JAK I	ns		ns		542.92	Y
AZ 960		JAK I	373	N	390.16	N	371.03	Y
SB1317	Zotiraciclib	JAK I/CDK I	355.6	Y	461.27	Y	422.11	Y
AZD7772		chK I	ns		777	Y	309.93	Y
LY2603618	Rabusertib	chK I	ns		ns		934.34	Y
CHIR-124		chK I	960.17	Y	652.17	Y	301.9	Y
KX2-391	Tirbanibulin	SRC I	466.1	Y	185.14	Y	32.6	Y
PD-166285		SRC/FGFR I	3000	ns	1220.8	nt	867.2	nt
Birinipant		IAP I	322.95	N	294	Y	ns	
KG-5		PDGFR I	2005.1	ns	1237.2	nt	941.7	nt
Hypericin		multiK I	114.6	N	115	N	115.6	N

Legend: ^1^ Cayman; ^2^ Medchem Express; ^3^ Focus Biomolecules; ^4^ Selleckchem; ns: not sensible; nt: not tested; Y: yes; N: no; I: inhibitor; CDK: Cyclin Dependent Kinase; PLK: Polo-like Kinase; Hsp90: heat shock protein 90; JAK: Janus Kinase; ChK: Checkpoint Kinase; FGFR: Fibroblast Growth Factor Receptor; IAP: Inhibitor of Apoptosis; MultiK: multikinase. In apoptosis assay, black refers to a concentration of 100 nM, green color refers to a concentration of 500 nM, and blue color refers to a concentration of 1000 nM.

## Data Availability

The data presented in this study are available in Table 1 and Supplementary Figure S1, Tables S1 and S2.

## Supplementary Materials

The following supporting information can be downloaded at: https://www.mdpi.com/article/10.3390/cancers14061575/s1, Figure S1: Kinase Inhibitor Library; Table S1: Variants found in CJM cell lines in relevant genes; Table S2: Hits Assignment.
