# Supplementary material for: Identification of New Vulnerabilities in Conjunctival Melanoma Using Image-Based High Content Drug Screening
Source: Cancers (Basel). 2022 Mar 19;14(6):1575. doi: 10.3390/cancers14061575 (PMC8946509; doi:10.3390/cancers14061575)
Supplement: Supplementary file 1 [file cancers-14-01575-s001.zip › Supplementary Figure S1 .pdf]

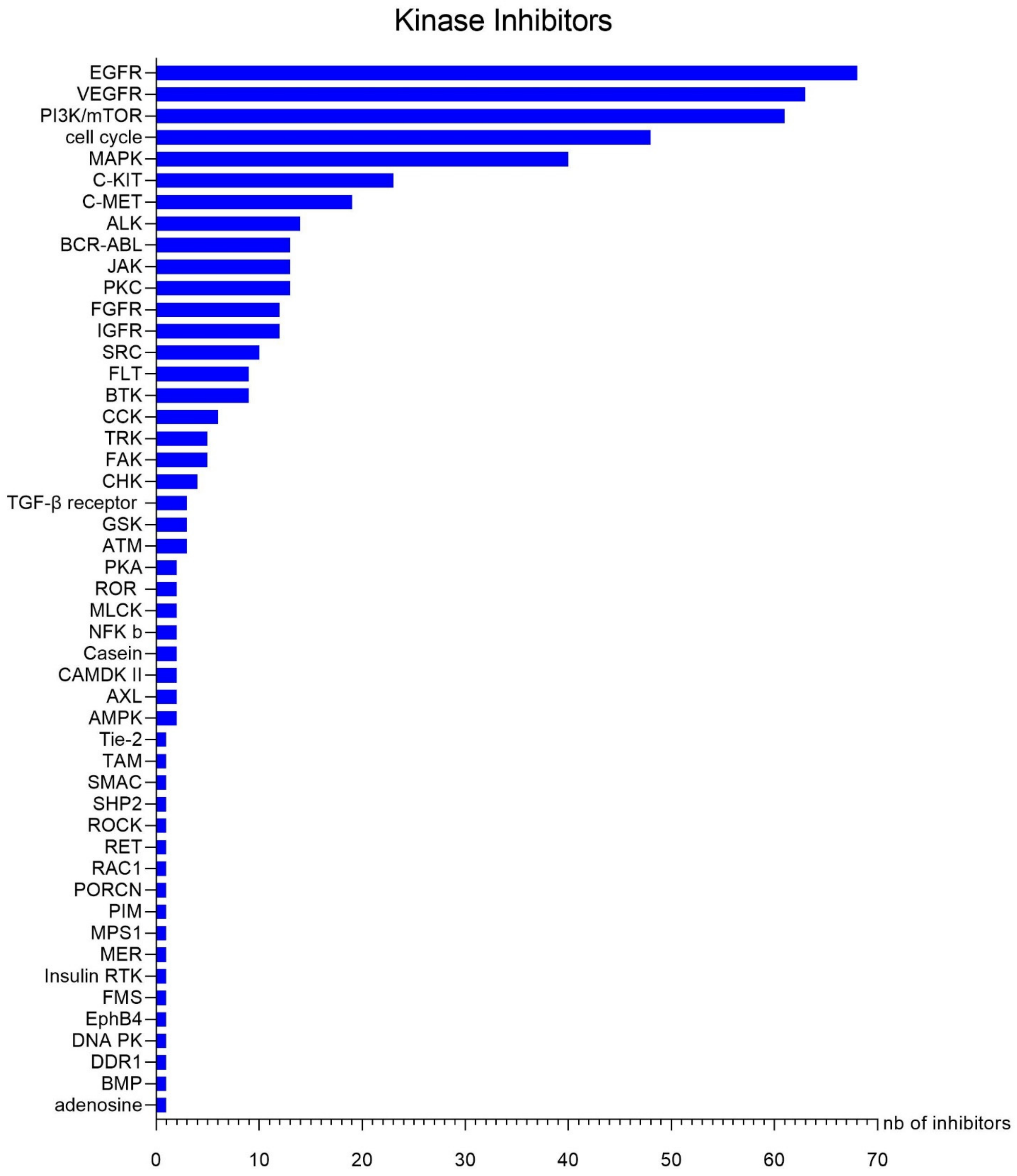

Supplementary Figure S1: Kinase Inhibitor Library

Many inhibitors targeted EGFR, VEGFR, PI3K/mTOR pathway, cell cycle or MAPK pathway.
